# Clean energy-led tourism development in Malaysia: Do environmental degradation, FDI, Education and ICT matter?

**DOI:** 10.1016/j.heliyon.2023.e21779

**Published:** 2023-11-04

**Authors:** Md Qamruzzaman

**Affiliations:** School of Business and Economics, United International University, Dhaka, Bangladesh

**Keywords:** Tourism, Clean energy, Environmental sustainability, FDI, ICT, Liner ARDL, Nonlinear ARDL

## Abstract

Concerning tourism, two lines of evidence are available in the literature, i.e., tourism's impact on macro fundamentals and determinants of tourism development. Regarding determinants, researchers have documented positive and negative associations between selected macro fundamentals and tourism development. The study's objective is to examine the impact of clean energy, foreign direct investment (FDI), education, and information and communication technology (ICT) on tourism development in Malaysia from 1990 to 2021. The study employed several econometrical techniques in investigating the empirical nexus, including the Bayer-Hancked cointegration test, ARDL Bound testing, Nonlinear ARDL, Toda-Yamamoto causality, and Fourier TY causality test. Moreover, the study employed dynamic OLS, Fully-modified OLS and CCR for the coefficient robust test. The study indicates that the utilization of renewable energy sources has the potential to alleviate the adverse environmental impacts associated with conventional energy sources. This, in turn, can enhance the appeal of tourist destinations to environmentally conscious visitors. Clean energy sources can provide tourism enterprises with cost-saving opportunities, improving profitability and ensuring long-term sustainability. Furthermore, the study reveals a noteworthy correlation between foreign direct investment (FDI) and tourism development. This implies that FDI plays a significant role in fostering tourism activities within the economy. Moreover, it has been found that education plays a crucial role in fostering tourism growth by positively influencing the quality of services and experiences offered to travelers. Finally, the study emphasizes the positive impact of information and communication technology (ICT) on the growth and advancement of the tourism industry. This is particularly evident in utilizing online booking systems, mobile applications, and immersive virtual tourism experiences.

## Highlights

• The present study investigates the effects of clean energy, environmental sustainability and FDI on tourism development.
• The study implemented ARDL, NARDL and Fourier TY causality tests …
• The study documented a positive linkage between clean energy, FDI and tourism development and carbon emission exposed adverse associations.
• The asymmetric assessment revealed asymmetric association both in the long-run and short-run.
• Directional causality revealed unidirectional causality between clean energy and Tourism and bidirectional association between environmental sustainability, FDI and tourism development.

## 1 Background of the study

The tourism industry is a multifaceted sector that draws knowledge from various fields and involves diverse individuals [[1], [2], [3]]. Extensive literature emphasizes the potential benefits of tourism in terms of job creation and overall economic growth, particularly in developing economies [[4], [5], [6], [7], [8]]. The concept of tourism-led growth suggests that international tourism, through improved quality and efficiency of tourist operations and the utilization of economies of scale, can contribute to revenue generation and economic development [[9], [10], [11]]. Given tourism's significant direct and indirect effects on the economy, it has become a widely explored topic in recent economic literature. Studies conducted by Ref. [12] and Lean, Chong [13] have investigated the effects of tourism on the local economy. These studies have confirmed a long-term relationship between tourism and economic growth, supporting the tourism-led growth hypothesis. Additionally, Go and Ng [14] found that exchange rate appreciation stimulates international tourist arrivals, contributing to economic sustainability. Tourism profoundly impacts the social, economic, and infrastructural development of the countries it visits. It fosters global commerce, enhances productivity in local settings, and reduces unemployment rates. Statistics indicate that tourism accounted for twenty percent of employment in the past decade and is projected to create an additional one hundred million jobs in the next decade. The literature unanimously asserts that the tourism sector promotes overall economic growth by increasing output [5,15]. Both developing and developed nations recognize the potential of expanding the tourism sector as a source of economic growth. Numerous works highlight the importance of the tourism industry and its contribution to overall growth, supporting the “tourist-led growth” theory., [[16], [17], [18], [19]].

The literature review reveals that factors other than this paper's selected factors are closely connected to the development of clean energy-led tourism. Governance is crucial in shaping the regulatory framework for integrating clean energy [Sou and Vinnicombe [20]]. Economic growth is both a driver and an outcome of clean energy development, as it is driven by the potential economic benefits that come with it. The urgency for clean energy adoption is highlighted by the need to reduce carbon dioxide emissions and fossil fuel consumption, which significantly impact the environment [Guo, Zhao [21]]. The consumption of renewable energy resources is also essential for achieving sustainable practices. Economic growth and eco-innovation work together to drive the development of clean energy solutions and ensure progress [Chau, Lin [22]]. Environmental factors and globalization influence energy consumption and economic growth [Chen [23]]. The complexities of exchange rate reference add a layer of complexity to energy transitions. International tourism receipts (TR) and quality of life (QoL) are indicators of economic gains and overall development [Sarpong, Bein [24]]. Natural resources are crucial in sustainable tourism development [Kongbuamai, Bui [25]]. Extensive research on the impact of sustainable tourism development reveals its complex nature, encompassing tourism's effects on tourism and its socio-cultural, economic, and environmental dimensions [[26], [27], [28], [29]].

This study focuses on the significance of clean energy (CE, hereafter), environmental sustainability (ES, hereafter), information and communication technology (ICT, hereafter), education (EDU, hereafter) and foreign direct investment (FDI, hereafter) as determinants of tourism development (TD, hereafter)t. Firstly, integrating renewable energy sources and travel opens up exciting possibilities in energy tourism, also known as business travel. This sector includes attractions such as manufacturing facilities, cutting-edge technological developments, and power plants designed with the tourist market in mind [30]. Energy tourism, specifically industrial tourism, has been shown in the existing literature to accelerate energy demand in the economy [31]. For instance, Beer, Rybár [32] and Aydin [33] documented a positive and statistically significant link between renewable energy consumption and tourism development in Turkey. Secondly, the literature has extensively documented the adverse environmental effects of tourism [[34], [35], [36], [37]]. However, it has also established that a well-organized tourist industry can contribute to environmental protection by adopting eco-friendly transportation and technology, thereby improving environmental quality [38]. Paramati, Alam [10] prove that improved transportation infrastructure, such as better roads and railways, reduces CO2 emissions. Additionally, research by Bhattacharya, Inekwe [39] demonstrates that sustainable tourism policies enhance knowledge about protecting the natural environment. Thirdly, there has been a growing body of research on the relationship between tourism and foreign direct investment [[40], [41], [42], [43]]. The literature offers several reasons for this connection. Foreign investors can maximize the economic benefits of increased tourism by investing in infrastructure projects such as the development of hotels and airports [44]. The amount of foreign direct investment (FDI) is strongly correlated with the number of managers and company owners seeking new business prospects in host countries [45]. Sanford and Dong [40] found that an increase in international tourist arrivals positively impacts foreign direct investment.

As highlighted by several studies, clean energy is a crucial aspect of a comprehensive framework [Sarpong, Bein [24] &Guo, Zhao [21]]. The adoption of renewable energy sources not only addresses environmental concerns but also aligns with sustainability goals. Clean energy is not just a reaction to environmental degradation but a proactive approach to mitigate the ecological impact of tourism and promote a harmonious relationship between the industry and its surroundings. Streimikiene, Svagzdiene [46] posit that environmental degradation poses challenges and opportunities. Kongbuamai, Bui [25] stress the urgent requirement to mitigate the environmental impact of tourism, underscoring the significance of adopting clean energy solutions. Adopting renewable energy sources in the tourism industry allows stakeholders to address environmental consequences and contribute to sustainable development. Banga, Deka [47] & Sarpong, Bein [24] have conducted studies that support this concept, indicating that implementing clean technologies is beneficial. FDI is seen as a driver of economic growth and can infuse capital and expertise into the tourism industry, catalyzing clean energy integration [Yue, Zubair [48], Munir and Iftikhar [49]]. However, caution is needed to avoid negative consequences such as environmental degradation, economic disparities, and cultural erosion [Chen [23]; Resende-Santos [50] and Fauzel, Seetanah [51]]. Education adds a nuanced layer to the clean energy discourse. While educational tourism can foster environmental awareness and sustainable practices, it can also lead to overcrowding, cultural conflicts, and exploitation Camargo, Winchenbach [52]]. Therefore, a comprehensive understanding is needed to ensure educational initiatives enhance clean energy-led tourism development while avoiding unintended consequences. ICT plays a crucial role in the digital age, amplifying the reach of clean energy solutions and facilitating communication, marketing, and operational efficiency [Law, Leung [53]. It enables businesses to align their practices with sustainable goals and cater to a global audience. However, careful implementation is necessary to avoid potential environmental impacts associated with digitalization [[54], [55], [56], [57], [58], [59], [60], [61]].

This study contributes to academic discourse and practical policy considerations by analyzing the correlation between clean energy initiatives and tourism development. Additionally, it explores the potential consequences of environmental degradation, foreign direct investment (FDI), education, and information and communication technology (ICT). First, the study's emphasis on clean energy as a catalyst for tourism development is both timely and relevant. Given the growing global awareness surrounding environmental concerns and the importance of sustainable tourism practices, integrating clean energy sources presents a significant opportunity to attract environmentally conscious travelers. The study emphasizes the significance of establishing a positive connection between clean energy and tourism development. It underscores how Malaysia's dedication to renewable energy can augment its attractiveness as a responsible and forward-thinking tourism destination. Second, the study explores the intricate correlation between environmental degradation and the advancement of tourism. The presence of a negative coefficient linked to environmental sustainability (ES) serves as a poignant reminder that unregulated CO2 emissions have the potential to diminish the appeal of tourist destinations. The study highlights the importance of considering this aspect, emphasizing the need to balance economic growth and environmental preservation. It urges policymakers to prioritize sustainable practices that protect the natural beauty of Malaysia's landscapes. Third, the study's investigation into the impact of foreign direct investment (FDI) on tourism development enhances the depth of the analysis. The positive coefficient associated with foreign direct investment (FDI) indicates that international investments substantially impact the tourism industry's growth. This insight emphasizes the critical importance of economic collaboration in developing contemporary infrastructure and facilities. Additionally, it underscores the interconnectedness between economic prosperity and the appeal of tourism. The study highlights the significance of education and information and communication technology (ICT) in molding the tourism industry's framework. The presence of positive coefficients assigned to education and ICT indicates that a well-educated workforce and advancements in technology have a positive impact on the development of tourism. This aspect highlights the importance of investing in human capital development and embracing technological innovation to enhance visitor experiences and improve destination competitiveness. Fifth, the study findings have implications that extend beyond the borders of Malaysia collectively. In the pursuit of aligning economic aspirations with environmental stewardship, nations across the globe are actively exploring ways to leverage the adoption of clean energy as a catalyst for sustainable tourism growth. This study offers a valuable blueprint in this regard. The study thoroughly examines the factors contributing to environmental degradation, foreign direct investment (FDI), education, and information and communication technology (ICT). By delving into these intricate aspects, it effectively analyzes the intricacies of contemporary tourism development. Moreover, it provides valuable insights that can be a compass for policymakers, business stakeholders, and researchers in pursuing a sustainable and prosperous tourism industry.

The motivation of the study is to investigate the impact of clean energy, environmental sustainability ICT, education and FDI on tourism development in Malaysia from 1990 to 2021. The present study has implemented several econometrical tools for assessing the empirical nexus. The variable's stationary properties have been revealed with unit roots following the ADF, PP, KPSS, and Ng-Perron tests. The long-run cointegration has been assessed by employing the Bayer-Hancked combined cointegration test; the long-run and short-run coefficients of CE, ES, and FDI on TB have been explored through the following linear Pesaran, Shin [62] and Nonlinear framework following. Shin, Yu [63]. The causal association among the variables has been tested with the implementation of the TY casualty test and the Fourier -TY causality test following Nazlioglu, Gormus [64]; furthermore, the study has implemented the dynamic OLS, fully modified OLS and canonical cointegration regression (CCR). Our empirical investigation unveiled the long-run cointegration among the variables. Refers to the magnitudes of explanatory variables, the study documented the positive influence of CE and FDI and the adverse effects of carbon emission. Furthermore, the asymmetric relations between CE, ES, FDI and TD have been documented with nonlinear assessment.

The remaining structure of the paper is as follows: the survey of related literature focuses on the nexus between clean energy, environmental sustainability, FDI and tourism development displayed in Section II. Section III deals with the data and methodology of the study. The empirical model estimation and interpretation are available in Section IV. A discussion of the study is reported in Section V, and the conclusion and suggestions are exhibited in Section VI.

## 2 Literature review and hypothesis development

This section deals with the conceptual development through the literature survey focusing on the nexus between CE, ES, FDI and TD. For hypothetical development, the study segregated the literature survey dealing with explanatory variables and tourism development, i.e …, clean energy and tourism, environmental sustainability and tourism, and FDI and tourism, respectively. The summary of the literature survey is displayed in Table 1.

**Table 1:** Summary Literature survey.

Author	Sample (year)	Method s	CE	ED	FDI	EDU	ICT
Sou and Vinnicombe [20]	WGI	2SLS			-VE		
Tindale, Vicario-Modroño [65]	EU-15 (2020 and 2021)	OLS				-VE	
Guo, Zhao [21]	40 developing Asian countries (2000–2021)	VAR	+VE				
Chau, Lin [22]	China (1988–2020)	NARDL		-VE			
Chen [23]	China (1990–2020)	NARDL			mixed		
Yue, Zubair [48]	50 countries across (2009–2018)	OLS			+VE		
Irfan, Ullah [66]	China (2001Q1 to 2019Q4)	ARDL		-VE			
Baidoo, Agbloyor [67]	SSA (2000–2016)	DSUR			-VE		
Chandel [68]	Spain	ARDL		-VE			
Banga, Deka [47]	38 OECD (2008–2019)	Dynamic GMM	+VE				
Camargo, Winchenbach [52]	Mexico	Econometric models				-VE	
Machado Toffolo, Simoncini [69]	1851 tourists participated in Glocal Education (2016–2019)	Econometric methods				+VE	
Baloch, Shah [70]							
Streimikiene, Svagzdiene [46]	Tourist areas	Econometric models	+VE				
Munir and Iftikhar [49]	Bangladesh, India, Nepal, Pakistan, and Sri Lanka (1995–2019)	ARDL			+VE		
Tang [71]	61 countries	The threshold regression model				-VE	
Della Lucia, Dimanche [72]	Mexico	Econometric models				+VE	
Wassie [73]	Ethiopia (2001–2019)	empirical models		-VE			
Tomasi, Paviotti [74]	Higher Education Institutions (HEIs)	Econometric models				+VE	
Edelheim [75]	Selected tourists destinations	Econometric models				+VE	
Sarpong, Bein [24]	8 Southern African countries (1995–2017)	OLS	+VE				
Kongbuamai, Bui [25]	ASEAN countries (1995–2016)	ARDL	-VE				
Resende-Santos [50]	small island economy of Cape Verde (2000–2016)	Econometric models			+VE		
Fan, Liu [76]	Host areas	a utility maximization model		-VE			
Ben Jebli, Ben Youssef [77]	22 CSA (1995–2010)	VECM	+VE		+VE		
Happ and Ivancsó-Horváth [78]	EU (2010–2016)	New model					+VE
Fauzel, Seetanah [51]	Mauritius (1984–2014)	DVECM			-VE		
Ásványi, Juhász-Dóra [79]	Caribbean	Econometric models	+VE				
Buhalis and Amaranggana [80]	A number of tourism destinations.	Econometric models					+VE
Özşen [81]	North Cyprus, Famagusta	in-depth interview				-VE	
Belsoy, Korir [82]	Protected areas	Econometric models	mixed				

### 2.1 Clean energy and tourism development

Existing literature provides two pieces of evidence. The first is that tourism development is encouraged by using clean energy. For instance, the study of Banga, Deka [47] explores the influence of renewable energy use on tourism development, specifically its impact on greenhouse gas emissions. Encouraging tourism to adopt renewable energy can expedite the transition to sustainable energy sources, boost its contribution to the global energy supply, and mitigate greenhouse gas emissions. Sarpong, Bein [24] say that given its significant energy consumption, the tourism industry has a strong financial motivation to adopt renewable energy for improved efficiency and long-term cost savings. They also found tourism plays a leading role in implementing various sustainable energy solutions, including strategies like reducing aircraft weight, adopting biofuels and fuel-efficient taxing, and implementing energy-saving measures in hotels through the use. Renewable energy sources in tourism can enhance local communities' quality of life by reducing environmental pollution and promoting sustainable development. Findings suggested that the travel and tourism sector should aim to become carbon-neutral in the long run. The industry can achieve carbon neutrality and advantages by adopting renewable energy sources. A study by Guo, Zhao [21] exhibits utilizing clean and sustainable energy sources can fuel tourism, minimize ecological harm, and provide advantages for nearby communities. Additionally, the adoption of renewable energy can aid in the mitigation of environmental pollution and the decrease of greenhouse gas emissions [Ben Jebli, Ben Youssef [77] and Banga, Deka [47]]. The study explores the link between renewable energy and sustainable tourism in the Caribbean, highlighting its potential to mitigate environmental pollution. Ásványi, Juhász-Dóra [79] explore the link between renewable energy and sustainable tourism in the Caribbean, highlighting its potential to mitigate environmental pollution.

The second line of evidence suggested that tourism development aggravated the state of environmental degradation. Streimikiene, Svagzdiene [46] state that the growth of tourism has resulted in higher energy consumption, leading to environmental degradation. Tourism development has also caused excessive water usage, contributing to both water scarcity and environmental degradation. Furthermore, increased waste generation due to tourism development has further worsened environmental pollution and degradation. Kongbuamai, Bui [25] found tourism negatively impacts land use and habitats, leading to the loss of natural habitats and ecosystems. It also contributes to soil erosion, pollution (including air, water, and noise pollution), and the depletion of natural resources. Tourism can put additional pressure on endangered species and generate significant plastic waste. Agreeing with this author, Baloch, Shah [70] say uncontrolled and overcrowded tourist populations can also adversely affect the environment and strain infrastructure. Although Belsoy, Korir [82] found a mixed eventuality that tourism activities in protected areas can disrupt ecosystems and wildlife habitats, it can also create employment opportunities. Sustainable tourism practices can reduce these negative impacts, such as promoting responsible tourism, implementing waste management systems, and supporting conservation efforts [Streimikiene, Svagzdiene [46] & Baloch, Shah [70]].

H1 Clean energy inclusion prompts sustainable Tourism development

### 2.2 Nexus between environment and tourism

No evidence supports a positive relationship between environmental degradation and tourism. On the contrary, these papers suggest that uncontrolled tourism can lead to environmental degradation, harming the natural environment and wildlife. Wassie [73] stated engaging in tourism activities within protected areas can result in adverse effects such as harm to delicate ecosystems, disruption of wildlife, and deterioration of natural resources. Irfan, Ullah [66] and Fan, Liu [76] discovered that tourism-related activities, such as increased foot traffic and construction, can cause soil erosion and negatively impact the land's quality. They also said the presence of tourism can increase the stress on endangered species and their habitats, ultimately causing their populations to decrease even more. Moreover, when there are more tourists, there is also more waste being produced, especially plastic waste. This can cause significant and lasting harm to the environment when uncontrolled and overcrowded tourist populations can negatively impact the environment, causing a decline in service quality and putting pressure on infrastructure. Conveyed by Chau, Lin [22]. Chandel [68] found the overconsumption of natural resources, such as water and energy, by tourists and tourism-related activities can deplete local resources and strain the environment. This includes excessive water use for hotels, resorts, and recreational activities.

H2 environmental sustainably, that is, CO2 reduction accelerated tourism development

### 2.3 Foreign direct investment-led tourism

There are two sorts of evidence available in the current literature reviews. The first group supports the affinity, and the latter group contradicts. The proponents say FDI and tourism have a positive correlation because FDI can contribute capital, technology, and expertise to the tourism sector, resulting in economic growth and progress. Yue, Zubair [48] indicate that FDI can notably impact the growth and advancement of the tourism sector, resulting in economic progress and development. Munir and Iftikhar [49] also found FDI is a valuable source of funding and capital for the tourism sector, enabling the industry to enhance infrastructure, enhance tourism services, and expand facilities. They've also seen FDI in the tourism sector can generate new jobs and employment prospects in the destination country, resulting in favorable socio-economic outcomes such as lower unemployment rates and enhanced living standards for local populations. Another researcher, Chen [23], said FDI in the tourism sector could lead to the sharing of advanced technologies and management practices, resulting in improved quality of services, increased operational efficiency, and the stimulation of innovation. Ben Jebli, Ben Youssef [77] stated FDI plays a crucial role in promoting sustainable tourism practices through adopting eco-friendly technologies, efficient resource management, and responsible tourism initiatives, ultimately contributing to the long-term sustainability of the tourism industry.

Notwithstanding, the opponents have different findings. Resende-Santos [50] mentioned relying too heavily on FDI for tourism development can create an uncertain reliance on foreign investment, resulting in economic vulnerability and instability within the tourism sector. Chen [23] also found unregulated growth in the tourism industry, which can be accelerated by foreign direct investment, can cause detrimental effects on the environment, such as pollution, destruction of habitats, and the depletion of natural resources. Fauzel, Seetanah [51] documented that) driven tourism growth can result in an imbalanced distribution of advantages, as local communities and small enterprises are frequently marginalized and need to benefit from the progress in tourism. Sou and Vinnicombe [20] also stated unregulated augmentation in tourism, often supported by foreign direct investment, can harm local cultures, such as the erosion of cultural heritage and the commercialization of traditional practices. FDI in the tourism sector can lead to economic leakage, where the money made from tourism is transmitted back to foreign investors instead of remaining within the local economy (Baidoo, Agbloyor [67].

H3 inflows of FDI positively induced tourism development

### 2.4 For education led tourism

The nexus between education and tourism can be explained in two different viewpoints. The favorable association between these variables is justifiable. Machado Toffolo, Simoncini [69] uncovered that traveling can offer informal learning experiences that result in positive educational outcomes and improved environmental awareness. Developing a mindset focused on tourism education is crucial for boosting job opportunities, tackling current challenges, and advocating for sustainable practices in the tourism industry. Tomasi, Paviotti [74] also advocate that educational tourism can support local development by providing non-formal learning opportunities while traveling. Universities and higher education institutions can contribute by sharing knowledge and promoting sustainable practices that preserve biodiversity and cultural heritage, leading to economic growth and the preservation of local assets. Edelheim [75] also unveiled that access to good tourism education is essential for decreasing informal work in the industry. Education can give people the skills and qualifications they need to have better jobs in tourism, leading to more opportunities and stability for individuals and communities.

Although education can benefit tourism, it can also have negative consequences. Özşen [81] discerned educational tourism can adversely affect host communities by causing overcrowding, noise pollution, and cultural conflicts. Furthermore, it can commercialize local culture, diminishing traditional practices and customs to mere tourist spectacles. In a study, Tindale, Vicario-Modroño [65] discovered despite its potential to promote sustainable practices, educational tourism can also contribute to environmental degradation. The influx of tourists, although seeking knowledge, can inadvertently cause harm to the environment through activities like littering, habitat destruction, and disturbance of wildlife. Similarly, Tang [71] said educational tourism has the potential to foster a heavy reliance on tourism as the main economic driver, resulting in a dependence on tourism for income generation. However, this over-reliance can lead to economic instability and susceptibility to external factors like natural calamities or economic downturns. Camargo, Winchenbach [52] unveiled educational tourism may exploit tourism workers. Education provides formal training and credentials; however, incredibly informal and low-skilled workers can be used without adequate regulation and enforcement. Education can benefit tourism, but it may additionally harm it.

Hypothesis: Education fosters Trousuim contribution to the economy.

### 2.5 For ICT-led tourism

The present body of literature encompasses two distinct categories of evidence. The first group discussed the positive nexus. The study by Law, Leung [53] expressed that the advancement of ICT has enhanced the communication between tourists and tourism businesses, resulting in improved customer service and satisfaction. This is exemplified by the utilization of online booking systems, catboats, and social media platforms, which enable tourists to engage with businesses instantly, facilitating the process of trip planning and reservation. Additionaly, Happ and Ivancsó-Horváth [78] discovered ICT has dramatically transformed the conduction of tourism marketing. Tourism businesses can effectively and efficiently promote their offerings to a worldwide customer base using social media platforms, online advertising, and search engine optimization (SEO). Car, Stifanich [83] found ICT has improved tourism operating efficiency, lowering costs and increasing production. Hotel management systems, revenue management software, and online payment systems have streamlined business processes, reduced manual labor, and improved precision. Another finding by Qiao, Ding [84] was the use of ICT has expanded the accessibility of tourism to a broader audience, including individuals with disabilities. Additionally, Buhalis and Amaranggana [80] found ICT has facilitated the collection and analysis of extensive data in the tourism industry, resulting in enhanced decision-making and improved customer experiences. This is exemplified by the ability of data analytics to provide insights into customer preferences, detect patterns, and tailor marketing strategies accordingly.

Hypothesis: ICT is positively associated with Tourism development.

## 3 Material and methods

**Fig. 1:**
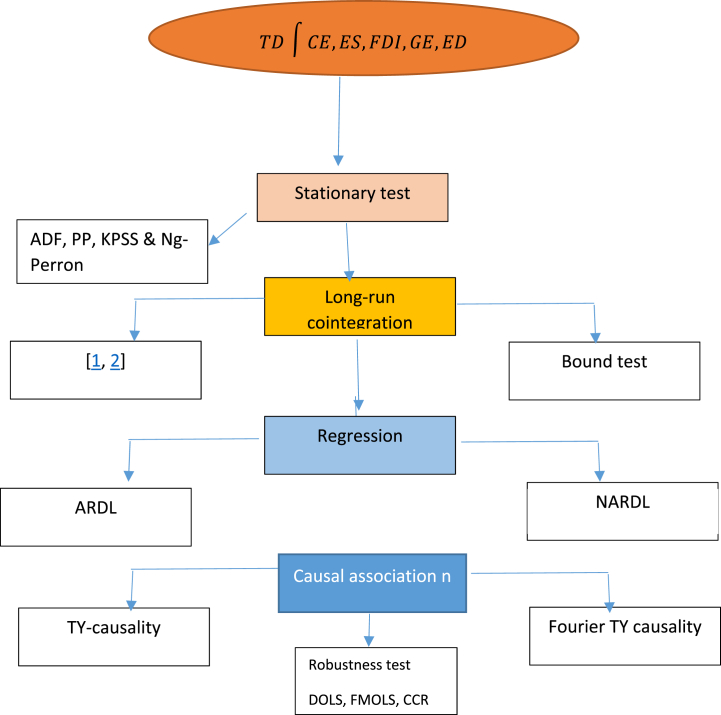
Estimation process.

To get a head start on accomplishing the purpose of our research, we have gathered the necessary information on the variables of interest. This research then described the empirical model by referring to previous research. After that, the defined empirical model has to be examined using various advanced econometric methods to get trustworthy output for forming policy. Therefore, the first step of the investigation is to evaluate the stationary property of the time series data. The research verified the long-term connection of the given model after it had first established the integration order of the variable. After that, to derive the policy measures, the long-term and short-term impacts of the explanatory factors on the outcome variables were investigated. Utilizing the right econometric technique allowed for the additional verification of the robustness of the long-run finding. The process of conducting empirical experiments using the appropriate instruments at each step of the study is outlined in Fig. 1. The following sections provide a comprehensive explanation of each approach that was considered.

### 3.1 Data

The study considered annual time series data for 1990–2018 for empirical stand validation. The motivation of the Study is to assess the role of clean energy, environment sustainability, inward FDI and governance quality on tourism development in the Malaysian economy for the period. All the pertinent data were extracted from the World Development Indicator (WDI), TC data published by the World Bank, and the World Governance Index (WGI). Table 2 exhibits the measurement of the variables (see Table 3).

**Table 2:** Proxy variables.

Variables name	Symbol	Measures	Sources	expected sign
Tourism development	TD	Tourism contribution to GDP	TCdata (WDI)	
Clean energy	CE	Renewable energy consumption		+
Environmental sustainability	ES	Metric tons per capita	WDWDII	–
Inflows of FDI	FDI	Net inflows of FDI as % GDP	WDI	+
Information and communication technology	ICT	Investment in ICT	OECD	+
Education	ED	Secondary school enrollment	WDI	+

**Table 3:** The null hypotheses for all three tests are defined as follows.

Cointegration test	Null hypothesis	Alternative hypothesis
F-bound test	$\gamma_{1}=\gamma_{2}=\gamma_{3}=\gamma_{4}=\gamma_{5}=\gamma_{6}=0$	Any, $\gamma_{1},\gamma_{2},\gamma_{3},\gamma_{4},\gamma_{5},\gamma_{6}\neq 0$
a *t*-test on the lagged dependent variable	$\gamma_{1}=0$	$\gamma_{1}\neq 0$
F-test on the lagged independent variable	$\gamma_{2}=\gamma_{3}=\gamma_{4}=\gamma_{5}=\gamma_{6}=0$	Any, $\gamma_{2},\gamma_{3},\gamma_{4},\gamma_{5},\gamma_{6}\neq 0$

### 3.2 Theoretical development and model specification

Existing literature dealing with the determinants of tourism development, the present study intends to explore the effects of clean energy, environmental sustainability, and inflows of FDI on tourism development in Malaysia. Based on the literature, the generalized relationship between CE, ES, FDI and TD, along with the control variables, is as follows:

(1) TD∫CE,ES,FDI,ICTEDu

with the mediating effects of clean energy, i.e., (CE*ES, CE*FDI), the above equation has been enlarged and represented as follows:

(2) TD∫CE,ES,FDI,(C*ES),(CE*FDI),ICTEDU

TD denotes tourism development, CE for clean energy, and ES explains environmental sustainability, FDI for foreign direct investment, GE for governmental effectiveness, and ED for education. Moreover, the mediating role of clean energy through ES and FDI on TD has been addressed with the inclusion of interactive terms. The above equations (1), (2) can reproduce in the following manner after log operation.

(3) $${TD}_{t}={\alpha_{0}+\beta }_{1}{CE}_{t}+\beta_{2}{ES}_{t}+\beta_{3}{FDI}_{t}+\beta_{4}{ICT}_{t}+\beta_{5}{EDU}_{t}$$

(4) $${TD}_{t}=\alpha_{0}+\gamma_{1}{CE}_{t}+\gamma_{2}{ES}_{t}+\gamma_{3}{FDI}_{t}+\gamma_{4}{\left(ES*CE\right)}_{t}+\gamma_{5}{\left(FDI*CE\right)}_{t}+\gamma_{6}{ICT}_{t}+\gamma_{7}{EDU}_{t}$$

where $\alpha_{0}$ is constant, the coefficient of $\beta_{1}\ldots \ldots \ldots \ldots \beta_{5}$ explain the relations of CE, ES, FDI, GE and ED on tourism development. Furthermore, the interactive effects can be found with the coefficient of $\gamma_{4}\;and\;\gamma_{5}$.

Using renewable energy means drawing power from a non-exhausting source, such as the sun or the wind, and using it again without causing long-term damage to the planet [85]. Countless renewable energy farms have sprung up throughout the globe in recent years to alleviate the energy issue in various nations. Ecotourism is tourism focusing on the environment to substantially benefit local populations [86]. Ecotourism has emerged as one of the fastest-growing sectors of the international tourism business. Because of the renewable energy sources, such as the installed solar panels and dish systems [87], there was an increase in the number of tourists who visited the area, which led to an increase in the tourist value proposition for the environment. Ecotourism and other forms of green tourism increasingly recognize the green energy sectors as a significant draw for tourists to visit the region [88]. In terms of existing literature following clean energy effects, it is anticipated that the inclusion of clean energy in the economy will boost the tourism contribution, suggesting a positive association; in other words $\beta_{1}=\frac{TD}{CE}>0$.

Environmental protection is essential for sustainable economic growth, and it is feasible to manage environmental deterioration by reducing carbon intensity. The ongoing release of greenhouse gases has publicly jeopardized the possibility of equal growth, particularly for developing countries. The regulated carbon emission hurt their overall production; consequently, they have shown little interest in regulating the carbon emission … Thus, it is assumed that carbon emission has a negative association with tourism development; in other words, $\beta_{2}=\frac{TI}{es}<0$. The nexus between tourism and FDI has produced two directional pieces of evidence. First, many researchers have postulated tourism as a determinant of inflows of FDI in the economy [[89], [90], [91]]. Foreign direct investment (FDI) may catalyze tourism, as pointed out by Haley and Haley (1997). Foreign direct investment (FDI) may be difficult due to the host country's distinctive economic, cultural, and political norms, but investors can mitigate these risks by researching in-country. One further reason to believe there is a direct link between foreign direct investment and visitor numbers is that FDI helps fuel economic growth that directly benefits the tourism industry (i.e., new tourist attractions and venues). Foreign direct investment (FDI) focusing on exports and increasing commerce increases consumers' and tourists' familiarity with the region's products and services. In directional causal association, several studies have revealed the bidirectional association between tourism and inflows of FDI [6]. Taking account of the existing nexus between tourism and FDI, it is anticipated that positive effects from FDI on tourism development; in other words $\beta_{3}=\frac{TD}{CE}>0$. Studies like Forsyth and Dwyer (2003) argued that foreign investment and know-how are paramount in creating and upgrading tourism-related infrastructure and fostering additional investment in the tourism sector. As such, a positive coefficient is expected in the present instance.

Unit root test:

In an empirical model estimate that considers time series data, analyzing the variables' qualities is necessary for choosing suitable techniques for determining the nexus between dependent and explanatory variables [92]. The study used several unit root tests to evaluate the stationarity qualities of the variables. These unit root tests included the ADF test [93], the P=P test [94], the DF-GLS test [95], the KPSS test [96], and the Z-A test [76] for one structural break in the research unit.

The ADF test has explored the stationary qualities using the lagged difference form of the target variable to solve the serial correlation issue. It is important to consider the following aspects of the system:

(5) $$\Delta Y=\gamma_{0}+\gamma_{1}Y_{t-1}+{y2}^{t}+\sum_{i=1}^{w}\alpha_{i}{\Delta Y}_{t-1}+\mu_{t}$$

Elliott and Rothenberg [73] have expanded the ADF test, which is still known as DF-GLS and is based on the ordinary least square calculation method (OLS). The stationary test provided by GF-GLS makes it possible to evaluate trends linearly, as seen in the following:

(6) $${\Delta y}_{t}^{d}=\alpha y_{t-1}^{d}+\sum_{i=1}^{p}\vartheta_{j}{\Delta y}_{t-i}^{d}+\sigma_{t}$$

where $y_{t}^{d}$ for de-trend data and $\sigma_{t}$ Stands for the white noise error term.

Elliott, Rothenberg [95] have developed an improved version of the ADF test, which is still referred to as DF-GLS and is based on the technique of computation known as ordinary least squares (OLS). Because GF-GLS offers a stationary test, it is now able to examine trends linearly, as can be seen in the following:

(7) $$y_{t}=\beta_{0}+\beta_{1}t+\gamma_{t}+\epsilon_{t}$$

(8) $$\gamma_{t}=\gamma_{t-1}+\theta_{t}$$

${where\;\beta }_{0}$ and $\beta_{1}$ Explain the deterministic term in constant form and a linear trend in equation (3), whereas, $\gamma_{t}$ Stands for the random walk factors in the estimation. Kwiatkowski, Phillips [96] proposed the following LM test statistics for stationary tests.

(9) $$LM=\frac{1}{T^{2}}\frac{\sum_{t-1}^{T}M_{t}^{2}}{\hat{\delta^{2}}}$$

Where, $M_{T}=\sum_{i=1}^{t}e_{i}$ stands for residuals from OLS estimation and $\delta^{2}$ is the variance estimator, which, under the null hypothesis, may remove nuisance parameters from the asymptotic distribution of the Lagrange multiplier (LM) statistic.

### 3.3 Bayer-combined cointegration test

The study implemented the cointegration test by following the framework proposed by Bayer and Hanck [97], commonly known as the combined cointegration test. The proposed cointegration test consists of four conventional tests of cointegration familiarized by Banerjee, Dolado [98], Peter Boswijk [99], Johansen [100], and Engle and Granger [101] with the null hypothesis of a no-cointegration test, the following Fishers' equation is considered in deriving the test statistics for detecting long-run association.

EG−JOH=−2[LN(PEG)+LN(PJOH)]

EG−JOH−BO−BD=−2[LN(PEG)−ln(PJPH)+ln(PBO)+ln(PBDM)]

where PBDM, PBO, PJOH, and PEG stand for the significance levels of Banerjee, Dolado [98], Boswijk [102], Johansen [100], and Engle and Granger [103], respectively.

Following Pesaran, Shin [62], the generalized ADRL model for the study was considered for detecting long-run and short-run coefficients by performing the following equation.

(10) $${\Delta lnTD}_{t}=\alpha_{0}+\sum_{i=1}^{n}\mu_{1}{\Delta lnTD}_{t-i}+\sum_{i=0}^{n}\mu_{2}{\Delta lnCE}_{t-i}+\sum_{i=0}^{n}\mu_{3}\Delta \ln\;{ES}_{t-i}+\sum_{i=0}^{n}\mu_{4}\Delta \ln\;{FDI}_{t}+\sum_{i=0}^{n}\mu_{5}\Delta \ln\;{GE}_{t-i}+\sum_{i=0}^{n}\mu_{6}{\Delta lnED}_{t-i}+\gamma_{1}{lnTD}_{t-i}+\gamma_{2}{lnCE}_{t-1}+\gamma_{3}{lnES}_{t-1}+\gamma_{4}\;\ln\;{FDI}_{t-1}+\gamma_{5}{lnGE}_{t-1}+\gamma_{5}{lnED}_{t-1}+\omega_{1t}$$

(11) $${\Delta lnTD}_{t}=\alpha_{2}+\sum_{i=1}^{n}\beta_{1}{\Delta lnTD}_{t-i}+\sum_{i=0}^{n}\beta_{2}{\Delta lnCE}_{t-i}+\sum_{i=0}^{n}\beta_{3}\Delta lnES+\sum_{i=0}^{n}\beta_{4}\Delta \ln\;{FDI}_{t}+\sum_{i=0}^{n}\beta_{5}\Delta \ln\;{GE}_{t-i}+\sum_{i=0}^{n}\beta_{5}\Delta \ln\;{ED}_{t-i}+\rho {ECT}_{t-1}+\omega_{1t}$$

For interactive terms:

(12) $${\Delta lnTD}_{t}=\alpha_{0}+\sum_{i=1}^{n}\mu_{1}{\Delta lnTD}_{t-i}+\sum_{i=0}^{n}\mu_{2}{\Delta lnCE}_{t-i}+\sum_{i=0}^{n}\mu_{3}\Delta \ln\;{ES}_{t-i}+\sum_{i=0}^{n}\mu_{4}\Delta \ln\;{FDI}_{t}+\sum_{i=0}^{n}\mu_{5}\Delta \ln\;{\left(ES*CE\right)}_{t-i}+\sum_{i=0}^{n}\mu_{6}\Delta \ln{\left(FDI*CE\right)}_{t}+\sum_{i=0}^{n}\mu_{7}\Delta \ln\;{GE}_{t-i}+\sum_{i=0}^{n}\mu_{8}{\Delta lnED}_{t-i}+\gamma_{1}{lnTD}_{t-i}+\gamma_{2}{lnCE}_{t-1}+\gamma_{3}{lnES}_{t-1}+\gamma_{4}\;\ln\;{FDI}_{t-1}+\gamma_{5}{\ln\left(ES*CE\right)}_{t-1}+\gamma_{6}\;\ln{\left(FDI*CE\right)}_{t-1}+\gamma_{7}{lnGE}_{t-1}+\gamma_{8}{lnED}_{t-1}+\omega_{1t}$$

(13) $${\Delta lnTD}_{t}=\alpha_{0}+\sum_{i=1}^{n}\mu_{1}{\Delta lnTD}_{t-i}+\sum_{i=0}^{n}\mu_{2}{\Delta lnCE}_{t-i}+\sum_{i=0}^{n}\mu_{3}\Delta \ln\;{ES}_{t-i}+\sum_{i=0}^{n}\mu_{4}\Delta \ln\;{FDI}_{t}+\sum_{i=0}^{n}\mu_{5}\Delta \ln\;{\left(ES*CE\right)}_{t-i}+\sum_{i=0}^{n}\mu_{6}\Delta \ln{\left(FDI*CE\right)}_{t}+\sum_{i=0}^{n}\mu_{7}\Delta \ln\;{GE}_{t-i}+\sum_{i=0}^{n}\mu_{8}{\Delta lnED}_{t-i}+\rho {ECT}_{t-1}+\omega_{1t}$$

Pesaran, Shin [62] and Sam, McNown [104] presented a unique set of asymptotic critical values in their research articles. The first batch included regressors with an I (1) level, whereas the second group had regressors with an I (0) level. The “no long-run association” null hypothesis could not be rejected if the value of the F-test statistic was less than the lower limit critical value or if the test statistic's absolute value of the *t*-test statistic was less than the absolute lower limit critical value.

### 3.4 Asymmetric ARDL estimation

In the recent literature, the application of asymmetric framework has been extensively used in effective policy formulation [[105], [106], [107], [108]]. The asymmetric framework assists in exploring the elasticity of explanatory variables through the decomposition of positive and negative shocks, which reveals fresh evidence over conventional relations. The study considered a nonlinear framework following Shin, Yu [109] in empirical assessment for detecting the asymmetric impact of economic policy uncertainty and financial inclusion on remittances. For gauging the asymmetric effects of CE, ES and FDI on TD, the following generalized equation is to be implemented:

(14) $${TD}_{t}={(\beta }^{+}{CE}_{1,t}^{+}+\beta^{-}{CE}_{1,t}^{-})+{(\gamma }^{+}{ES}_{1,t}^{+}+\gamma^{-}{ES}_{1,t}^{-})+{(\pi }^{+}{FDI}_{1,t}^{+}+\pi^{-}{FDI}_{1,t}^{-})+\delta_{i}X_{t}+\varepsilon_{t}$$

where $\beta^{+},\beta^{-},\gamma^{+},\gamma^{-}\text{And}\;\text{Stands}\;\text{for}$ the long-run pavements. The coefficient of $\beta^{+}\;\text{and}\;\beta^{-}$ specifies the effect of positive and negative shocks in CE, ES, and $\gamma^{+}\text{and}\;\gamma^{-}$ Denotes the asymmetric effects of FDI on RE. Furthermore, the coefficients of $\delta_{i}$ Measures the effects of control variables in the equation.

The asymmetric shock of clean energy, i.e., CE^+^; CE^−^, environmental sustainability, i.e., ES^+^; ES^−,^ and Foreign Direct Investment (FDI^+^; FDI) can be derived in the following manner.

$$\left\{ \begin{array}{c} {POS\left(CE\right)}_{1,t}=\sum_{k=1}^{t}{lnCE}_{k}^{+}=\sum_{K=1}^{T}\mathrm{MAX}\left({\Delta lnCE}_{k},0\right)\\ {NEG\left(CE\right)}_{t}=\sum_{k=1}^{t}{lnCE}_{k}^{-}=\sum_{K=1}^{T}\mathrm{MIN}\left({\Delta lnCE}_{k},0\right) \end{array}\right.:\begin{array}{c} {POS\left(ES\right)}_{1,t}=\sum_{k=1}^{t}{lnES}_{k}^{+}=\sum_{K=1}^{T}\mathrm{MAX}\left({\Delta lnES}_{k},0\right)\\ {NEG\left(ES\right)}_{t}=\sum_{k=1}^{t}{lnES}_{k}^{-}=\sum_{K=1}^{T}\mathrm{MIN}\left({\Delta lnES}_{k},0\right) \end{array}$$

$$;\begin{array}{c} {POS\left(FDI\right)}_{1,t}=\sum_{k=1}^{t}{lnFDI}_{k}^{+}=\sum_{K=1}^{T}\mathrm{MAX}\left({\Delta lnFDI}_{k},0\right)\\ {NEG\left(FDI\right)}_{t}=\sum_{k=1}^{t}{lnFDI}_{k}^{-}=\sum_{K=1}^{T}\mathrm{MIN}\left({\Delta lnFDI}_{k},0\right) \end{array}$$

Now, equation (14) is transformed into asymmetric long-run and short-run coefficient assessment as follows:

(15) $${\Delta TD}_{t}=\partial U_{t-1}+{(\beta }^{+}{CE}_{1,t-1}^{+}+\beta^{-}{CE}_{1,t-1}^{-})+{(\gamma }^{+}{ES}_{1,t-1}^{+}+\gamma^{-}{ES}_{1,t-1}^{-})+\left(\pi^{+}{FDI}_{1,t-1}^{+}+\pi^{-}{FDI}_{1,t-1}^{-}\right)+\delta X_{1,t-1}^{*}+{\sum_{j=1}^{m-1}\lambda_{j}{\Delta TD}_{t-j}}_{0}+\sum_{j=1}^{n-1}{(\pi }^{+}{\Delta CE}_{1,t-1}^{+}+\pi^{-}{\Delta CE}_{1,t-1}^{-})+\sum_{j=0}^{m-1}{(\pi }^{+}{\Delta ES}_{1,t-1}^{+}+\pi^{-}{\Delta ED}_{1,t-1}^{-})+\sum_{j=0}^{m-1}{(\beta }^{+}{\Delta FDI}_{1,t-1}^{+}+\beta^{-}{\Delta FDI}_{1,t-1}^{-})+\sum_{j=0}^{m-1}\mu \Delta X_{1,t-1}^{*}+\varepsilon_{t}$$

## 4 Estimation and interpretation

### 4.1 Unit root test

To document the stationary properties, the present study has implemented a unit root test following Dickey and Fuller [93] Phillips and Perron [94], Elliott, Rothenberg [95] with the null hypothesis of not stationary and Kwiatkowski, Phillips [96], with the null hypothesis of stationary. Referring to the results in Table 4, the study established that all the variables become stationary in the first difference, i.e., I (1).

**Table 4:** Results of the unit root test.

At level					After first difference			
	ADF	GF-DLS	PP	KPSS	ADF	GF-DLS	PP	KPSS
*For Brazil*								
TOR	−2.1226	−1.2022	−2.0951	0.6163***	−5.394***	−8.3868***	−9.5187***	0.0195
CE	−1.472	−1.6982	−2.54	0.9148***	−5.6605***	−7.4303***	−5.7189***	0.0189
FDI	−1.9376	−1.857	−1.9151	0.7589***	−9.4053***	−9.367***	−7.0486***	0.0211
EDU	−1.8533	−1.6246	−0.7433	0.7182***	−6.8334***	−9.5359***	−9.454***	0.0185
ICT	−1.4958	−2.3929	−2.2907	0.6236***	−8.6842***	−7.2028***	−5.4413***	0.0209
CO2	−2.1417	−1.1947	−1.6686	0.7985***	−8.0399***	−7.8238***	−7.8882***	0.0204

The study further executed the unit root test following Ng and Perron [110] to confirm the stationary test more efficiently, and the test statistics of MZa, MZt, MSB, and MPT are displayed in Table 5. Refers to the Ng-Perron unit root test, it is apparent that all the test statistics derived with a first difference are statistically significant at a 1 % significance level, establishing the rejection of the null hypothesis. Alternatively, established variables are stationary after the first difference.

**Table 5:** Shows the results of the Ng-Perron unit root test.

	At level				At first difference			
	MZa	MZt	MSB	MPT	MZa	MZt	MSB	MPT
TOR	−2.7022	−1.4075	0.36	9.1028	−21.118	−4.9452	0.1671	4.5898
CE	−1.6798	−0.7051	0.2306	8.0017	−17.769	−5.1961	0.1486	4.3281
FDI	−2.3912	−1.1652	0.2873	7.9229	−18.66	−5.3984	0.1364	3.4769
EDU	−2.6274	−0.8085	0.2295	7.9674	−17.107	−5.6955	0.1459	4.2468
ICT	−2.0821	−1.7101	0.2701	7.3077	−23.519	−5.3641	0.1677	3.6078
CO2	−1.7901	−1.2593	0.3354	7.2726	−24.234	−5.68	0.1558	4.9014

The study implemented a cointegration test following the framework offered by Bayer and Hanck [97] and Maki [111]. According to the test statistics exhibited in Table 6, the null of no-cointegration has been rejected, alternatively establishing the long-run association in the empirical equation.

**Table 6:** Shows the results of the Bayer-Hanck combined cointegration test.

		1		2	3	4	5
Panel A: Bayer-Hancked Combined Cointegration test							
EG-JOH		14.557		11.223	11.148	10.815	10.631
CV at 5 %		11.229		10.895	10.637	10.576	10.419
EG-JOH-BO-BDM		37.042		28.964	23.955	22.178	20.928
CV at 5 %		21.931		21.106	20.486	20.143	19.888
Panel B: Maki cointegration							
**Number of Breaks** **Points**	**Test Statistics**		**[Critical Values]**		**Break Points**		
Tb < 5							
0	−9.9144		−6.306		2006,2006,2012,2021,1998		
1	−7.0013		−6.494		1999,1995,2015,2000,1994		
2	−8.7739		−8.869		2003,2008,2005,2004,2013		
3	−8.2591		−9.482		2018,1997,2001,2017,1999		

The long-run assessment has been extended by implementing a standard Wald test under the symmetric and asymmetric framework. Table 7 reported the test statistics derived from the Wald test of F_overall_, t_DV_, and F_IDV,_ respectively. And found statistically significant at a 1 % level, confirming a long-run tie between explained and explanatory variables.

**Table 7:** Shows the results of the Symmetric and asymmetric cointegration test.

long-run cointegration	F_*overall*_	t_DV_	F_*IDV*_
ARDL	7.564***	−6.903***	8.869***
NARDL	9.479***	−6.851***	7.793***

### 4.2 Long-run assessment: the effects of CE, ES, FDI, GQ, and ED on TD

In the long run, the symmetric and asymmetric magnitudes of explanatory variables on tourism development are reported in Table 8, which includes model [97] for linear assessment and Model [112] for nonlinear assessment.

**Table 8:** Shows the results of long-run coefficients: symmetric and asymmetric assessment.

	Symmetric estimation						Asymmetric estimation				
	Coefficients	t-stat			Std. error			Coefficients	t-stat		Std. error
CE	0.1708	0.0103			16.5825		${CE}^{+}$	0.1723	0.0073		23.6027
ES	−0.0626	0.0095			−6.5894		${CE}^{-}$	0.178	0.0062		28.7096
FDI	0.0899	0.0082			10.9634		${ES}^{+}$	−0.1777	0.004		−44.425
EDU	0.0542	0.0043			12.6046		${ES}^{-}$	−0.1121	0.0099		−11.3232
ICT	0.1199	0.0055			21.811		${FDI}^{+}$	0.1635	0.0112		16.35
C	0.0617	0.0105			5.8669		${FDI}^{-}$	0.1754	0.0026		67.4615
							EDU	0.0632	0.0078		8.0697
							ICT	0.0944	0.0078		12.0181
							C	0.1107	0.0097		11.3743
$W_{LR}^{CE}$								6.696			
$W_{LR}^{ES}$								9.149			
$W_{LR}^{FDI}$								12.068			
Panel –A: Short-run coefficients											
CE	0.0312		0.0116	2.6896		${CE}^{+}$		*0.0396*	*0.0037*	*10.7027*	
ES	−0.0618		0.0027	−22.8888		${CE}^{-}$		*0.0081*	*0.0028*	*2.8928*	
FDI	0.0495		0.0102	4.8529		${ES}^{+}$		*−0.0333*	*0.007*	*−4.7571*	
EDU	0.0312		0.0116	2.6896		${ES}^{-}$		*−0.0347*	*0.0041*	*−8.4634*	
ICT	0.0618		0.0027	22.8888		${FDI}^{+}$		*0.0651*	*0.0056*	*11.625*	
ECT (-1)	−0.29123		0.010411	−27.9737		${FDI}^{-}$		*0.0212*	*0.0022*	*9.6363*	
						GQ		0.0298	0.008	3.725	
						ED		0.0289	0.004	7.225	
								−0.24684	0.010479	−23.5557	
$W_{SR}^{CE}$								8.428			
$W_{SR}^{ES}$								5.565			
$W_{SR}^{FDI}$								2.816			
Panel B: Residual Diagnostic test											
$x_{Auto}^{2}$	0.776							0.715			
$x_{Het}^{2}$	0.857							0.756			
$x_{Nor}^{2}$	0.693							0.586			
$x_{RESET}^{2}$	0.789							0.801			

For clean energy, the study documented a positive and statistically significant tie with TD in the long-run (β=0.1708:P<0.01) and short-run (β=0.0312:P<0.01). A positive coefficient about renewable energy (CE) signifies that an escalation in clean energy endeavors has a favorable impact on the advancement of tourism. This finding suggests that destinations that place a high value on and allocate resources towards sustainable energy sources, such as solar, wind, and hydropower, are expected to observe a rise in tourism expansion. Travelers who prioritize environmental sustainability are more inclined to choose destinations that exhibit a strong dedication to sustainable practices, particularly if they have implemented clean energy initiatives. Our study findings are supported by the existing literature Beer, Rybár [32].

The coefficient of Environmental Sustainability (ES) is found to be negative and statistically significant in the long-run (π=−0.0626.:P<0.01) and short-run (π=−0.0618:P<0.01), suggesting excessing CO2 dwindle the process of TD by 0.629 % (0.618) due to a 10 % increase of CO2 in the ecosystem. The negative tie between CO2 and TD is supported by the literature offered by Tong, Zhang [113]. Literature suggests that the elevated levels of carbon dioxide CO2 emissions have been found to hurt the progress of tourism development, as evidenced by the unfavorable coefficient associated with environmental sustainability (ES). This finding aligns with the concept that travel individuals tend to gravitate towards locations that offer high air quality and picturesque natural surroundings. As carbon dioxide emissions continue to increase, there is a growing concern regarding the potential environmental degradation that may ensue.

A positive statistically significant connection between FDI and TD was revealed in the long-run (π=0.0899.:P<0.01) and short-run (π=0.0495:P<0.01) assessment, implying the inflows of FDI in Malaysia foster the possibilities and contribution tourism industry in the aggregated economy. Precisely, a 10 % positive change in FDI could amplify TD in Malaysia by 0.899 % (0.495 %) in the long run (short-run). Moreover, the existing literature supports the study findings, such as Craigwell and Moore [44] and Moghavvemi, Ormond [114]. The study elaborates that a positive coefficient about foreign direct investment (FDI) signifies that the augmentation of FDI positively impacts tourism development. This finding implies that tourist destinations that attract higher levels of foreign investment are prone to witnessing enhanced infrastructure, improved amenities, and an overall increase in attractiveness. Infrastructure improvements have the potential to lead to a rise in the number of visitors and subsequently boost tourism revenue [[115], [116], [117]].

The coefficient of education (EDU) disclosed positively associated with TD in Malaysia in the l =0.0542:P<0.01) and short-run (π=0.0312:P<0.01), indicating the presence of a positive coefficient for education (EDU) suggests that a higher level of education among the local workforce has a beneficial impact on the development of tourism. An educated local population has the potential to offer travelers with exceptional services, experiences, and interactions. Furthermore, it is worth noting that educational institutions have the potential to play a pivotal role in cultivating proficient individuals who actively contribute to the growth and advancement of the tourism sector. Our study is in line with the literature offered by [[118], [119], [120], [121], [122]]

The study exposed a catalyst role of ICT in the process of augmenting the tourism development in Malaysia in the long-run (π=0.1199;P<0.01) and short-run (π=0.0618:P<0.01), suggesting advancements in technology have a positive effect on the development of tourism. Information and Communication Technology (ICT) advancements, such as the implementation of enhanced online booking systems, the development of user-friendly mobile applications for navigation purposes, and the provision of immersive virtual tourism experiences, can significantly augment and elevate the overall quality of the tourist experience. Our findings align with the existing literature [[123], [124], [125]]. The positive coefficient observed for ICT suggests that advancements in technology positively influence the development of the tourism industry [126]. Enhancing the overall tourist experience can be achieved by implementing improved online booking systems, developing mobile apps for navigation, and providing virtual tourism experiences, leading to enhanced tourism operations' effectiveness and appeal to technologically proficient travelers [127].

The empirical model passes several residual diagnostic tests to confirm the model's estimation robustness and efficiency. Refers to the test statistics, it is apparent that the tested models are free from serial correlation, have no issue with heteroskadacity, and are normally distributed error terms. Furthermore, the model stability has been confirmed with the REMSE test.

### 4.3 Mediating the role of clean energy through ES and FDI on TD

The following estimation deals with the extensions of the prime model with the inclusion of mediating effects of clean energy through the channel of environmental sustainability (CE*ES) and inflows of FDI (CE*FDI). The symmetric and asymmetric model results with interactive terms are displayed in Table 9.

**Table 9:** Results of symmetric and asymmetric estimation; mediating effects of clean energy.

Panel A: Long run cointegration							
		F_*overall*_	t_DV_	F_*IDV*_			
ARDL		12.761***	−6.588***	11.178***			
NARDL		13.245***	−5.508***	12.734***			
	Coefficient	t-stat	std. error				
Panel B: Long-run coefficients							
CE	0.1791	0.0019	94.2631	${CE}^{+}$	0.1732	0.0069	25.1014
ES	−0.1312	0.0117	−11.2136	${CE}^{-}$	0.141	0.0057	24.7368
FDI	0.092	0.0073	12.6027	${ES}^{+}$	−0.1796	0.0112	−16.0357
CE*ES	0.1226	0.0089	13.7752	${ES}^{-}$	−0.1202	0.0115	−10.4521
CE*FDI	0.071	0.0065	10.923	${FDI}^{+}$	0.1313	0.0043	30.5348
EDU	0.0566	0.0104	5.447	${FDI}^{-}$	0.1586	0.0074	21.4324
ICT	0.0317	0.0024	13.1064	${CE*ES}^{+}$	0.1046	0.0114	9.1754
C	−0.0945	0.0079	−11.8252	${CE*ES}^{-}$	0.1254	0.0042	29.8571
				${CE*FDI}^{+}$	0.178	0.0029	61.3793
				${CE*FDI}^{-}$	0.1297	0.008	16.2125
				EDU	0.183	0.006	30.5
				ICT	0.1902	0.0025	76.08
				C	−0.0726	0.0114	−6.3289
$W_{LR}^{CE}$					10.285		
$W_{LR}^{ES}$					5.759		
$W_{LR}^{FDI}$					10.605		
$W_{LR}^{CE*ES}$					9.485		
$W_{LR}^{CE*FDI}$					6.542		
Panel C: Short-run coefficients							
CE	0.0513	0.0117	4.3846	${\Delta CE}^{+}$	−0.0195	0.0101	−1.9306
ES	−0.0685	0.0073	−9.3835	${\Delta CE}^{-}$	−0.0073	0.0083	−0.8795
FDI	0.0655	0.0069	9.4927	${\Delta ES}^{+}$	−0.0242	0.0116	−2.0862
CE*ES	0.038	0.003	12.6666	${\Delta ES}^{-}$	−0.0489	0.0075	−6.52
CE*FDI	0.0149	0.0086	1.7325	${\Delta FDI}^{+}$	0.028	0.0086	3.2558
EDU	0.0617	0.0031	19.9032	${\Delta FDI}^{-}$	0.0389	0.0106	3.6698
ICT	0.0372	0.0114	3.2631	${\Delta CE*ES}^{+}$	0.0187	0.0057	3.2807
*ECT(-1)*	−0.7198	0.0155	−46.2320	${\Delta CE*ES}^{-}$	0.037	0.0118	3.1355
				${\Delta CE*FDI}^{+}$	0.0239	0.0114	2.0964
				${\Delta CE*FDI}^{-}$	0.0171	0.0117	1.4615
				EDU	0.022	0.0026	8.4615
				ICT	0.0231	0.0116	1.9913
				ECT (-1)	−0.24684	0.010479	−23.5557
Panel D: Symmetry test							
$W_{SR}^{CE}$					3.424		
$W_{SR}^{ES}$					7.335		
$W_{SR}^{FDI}$					3.745		
$W_{SR}^{CE*ES}$					8.349		
$W_{SR}^{CE*FDI}$					9.396		
Panel –E: Residual Diagnostic test							
$x_{Auto}^{2}$	0.865				0.681		
$x_{Het}^{2}$	0.822				0.819		
$x_{Nor}^{2}$	0.675				0.678		
$x_{RESET}^{2}$	0.68				0.889		

### 4.4 For symmetry

The study revealed that clean energy and the inflow of FDI positively connected with tourism development in Malaysia in the long and short run. On the other hand, the effect of carbon emission is negatively associated with tourism development in the long and short run. Concerning the mediating role of clean energy on tourism development through the channel of environmental sustainability and inflows of FDI, the study documented, both in the long-run and short-run, positive and statistically significant influences targeting tourism development. In particular, a 10 % progress in clean energy inclusion in the economy indirectly accelerates tourism development with the control of carbon emissions, alternatively ensuring environmental sustainability by 0.0151 % in the long run and by 0.0147 % in the short run. Moreover, a 10 % increase in clean energy integration assists tourism development through FDI promotion by 0.247 % and 0.282 % in the long and short run, respectively. Study findings advocate that the effects of clean energy on tourism development can be observed through direct and indirect channels. Thus, the energy transition from conventional to renewable sources might appear as an alternative to mitigate the impact of carbon emissions and promote foreign investors.

### 4.5 For asymmetry

Refers to the asymmetric coefficients of clean energy and inflows of FDI on tourism development both in the long-run and short-run the study documented a similar line of association aligned with the previous model assessment (see Table 6). Furthermore, the consistency disclosed for the asymmetric impact of environmental sustainability on tourism development with the prime model assessment for the long and short run. The asymmetric coefficients of interactive terms, i.e., ${\left(CE*ES\right)}^{+};{\left(CE*ES\right)}^{-}\&{\left(CE*FDI\right)}^{+};{\left(CE*FDI\right)}^{-}$, positive and statistically significant at 1 %, suggesting the contributory role of clean energy development on tourism development through the environmental development channel and foreign investors' participation in the economy. Particularly, a 1 % positive (negative) innovation produces the improvement (reduction) in tourism contribution by 0.1046 % (0.0846 %) through the channel of environmental correction and by 0.0733 % (0.0811 %) with the collaboration of FDI inflows in the economy.

### 4.6 Directional investigation: TY causality and Fourier TY causality

Next, the directional relationship between TD, ES, CE, GE, FDI, and EDU has been investigated by implementing the Fourier TY causality test offered by the TY causality test proposed by Toda and Yamamoto [128]. The results of the causality assessment displayed in Table 10 include Panel –A for Fourier TY causality and Panel –B for TY causality.

**Table 10:** Shows the results of Fourier TY and TY causality tests.

	TD	CE	ES	FDI	ICT	EDU	Causal association
Panel A: Fourier TY causality test							
TD		6.033**	1.321	4.25*	6.231**	2.229	FDI→TD; EDU←→TD; TD→ICT; CE→ES; FDI←→ES; ICT←→ES; EDU→ICT; FDI→EDU
CE	4.963*		2.036	1.725	5.963*	6.019**	
ES	6.986**	1.932		2.554	3.239	6.676**	
FDI	1.007	2.379	5.625*		6.89**	4.57*	
ICT	7.156**	6.352**	6.777**	1.715		5.668*	
EDU	1.005	7.069**	2.993	1.58	5.325*		
Panel B: TY causality test							
TD		4.025*	0.489	4.683*	0.822	6.253**	ES←→TD; FDI→TD; ICT←→TD; CE→ICT; ES→FDI; ES→ICT; EDU→FDI; FDI→ICT; EDU→ICT
CE	1.289		3.843	2.049	0.729	1.539	
ES	3.76	5.32*		1.788	0.359	4.455*	
FDI	4.966*	5.911*	0.629		3.023	2.335	
ICT	3.417	1.56	2.74	5.273*		4.85*	
EDU	3.775	5.222*	0.283	5.341*	6.78**		

According to the Fourier TY causality test, the Study documented a unidirectional causal association between education and tourism development [EDU←→TD], foreign direct investment and environmental sustainably [FDI←→ES]; and governance quality and environmental sustainability [ICT←→ES], respectively. Furthermore, several unidirectional causalities have been revealed during the assessment, particularly from foreign direct investment to tourism development [FDI→TD], tourism development to governance quality and clean energy to environmental sustainability. In terms of the TY causality test (see Panel –B), it is apparent that the feedback hypothesis holds in explaining the association between environmental sustainability and tourism development [ES←→TD] and governance quality and tourism development [ICT←→TD]. Additionally, the unidirectional tie unveiled for foreign direct investment to tourism development [FDI→TD]; environmental sustainability to foreign direct investment [ES→FDI]; education to foreign direct investment [EDU→FDI].

### 4.7 Coefficient robustness test: DOLS, FMOLS, and CCR

The study has extended the investigation by implementing dynamic QLS, Fully-modified OLS and CCR regression for robustness. The robustness results are displayed in Table 11, including Panel –A for empirical nexus without interactive term and Panel –B for the interactive term. Refers to the coefficients and sign of explanatory variables, the study confirmed the same line of association that clean energy and FDI positively influenced tourism and environmental sustainability; that is, carbon emission declined the tourism contribution to the economy. Furthermore, the coefficient of the interactive term (CE*ES) & (CE*FDI) on tourism revealed a positive association, implying the mediating role of clean energy.

**Table 11:** Results of robustness test: FMOLS, DOLS, and CCR long-run analysis.

Variables	Coefficient	std	t-stat	Coefficient	std	t-stat	Coefficient	std	t-stat
**Panel A: without interactive term**									
CE	−0.1448	0.0313	−4.6261	−0.1741	0.0379	−4.5936	−0.172	0.0361	−4.7645
ES	−0.1727	0.0258	−6.6937	−0.1822	0.0734	−2.4822	−0.1817	0.0747	−2.4323
FDI	0.0974	0.057	1.7087	0.2015	0.0358	5.6284	0.1591	0.0827	1.9238
GE	0.0573	0.0167	3.4311	0.0388	0.0401	0.9675	0.0794	0.0987	0.8044
ED	0.1104	0.0441	2.5034	−0.1541	0.0562	−2.7419	−0.1059	0.0452	−2.3429
**Panel B: With the interactive term**									
CE	−0.1557	0.0615	−2.5317	−0.165	0.0812	−2.032	−0.1468	0.0593	−2.4755
ES	−0.1784	0.0161	−11.0807	−0.1771	0.0572	−3.0961	−0.1778	0.0862	−2.0626
FDI	0.1064	0.0724	1.4696	0.1789	0.0577	3.1005	0.1911	0.0496	3.8528
GE	0.0371	0.048	0.7729	0.0975	0.0393	2.4809	0.0283	0.0317	0.8927
ED	0.1466	0.0669	2.1913	−0.0844	0.079	−1.0683	−0.0494	0.0666	−0.7417
CE*ES	0.0219	0.0757	0.2892	0.0368	0.0165	2.2303	0.0644	0.0969	0.6646
CE*FDI	0.0727	0.0175	4.1542	0.1112	0.0773	1.4385	0.1523	0.0513	2.9688

## 5 Discussion of the findings

In terms of clean energy coefficients, it is advocated that for sustainable and progressive development in tourism, it is imperative to encourage economic agents for an energy transition from fossil fuel to renewable energy. Eco-tourists prefer renewable energy sources since conventional energy sources are harmful to the environment and pose a threat to the local population when they are used. Examples include the possible dangers that nuclear energy poses to the area's environment and its stigma [129]. Ecotourism, also known as green tourism, is a style that is more responsible, more in tune with nature, and more helpful to the environment than other types of tourism. Ecotourism also goes by green tourism [85]. The positive association of our study findings is supported by existing literature [33,105,[130], [131], [132], [133]]. As substantiated by both linear and nonlinear assessments, clean energy has the potential to foster tourism development in Malaysia. The Malaysian tourism sector can benefit from the development of CE in the following manners: using renewable energy sources, such as solar, wind, and hydro, can significantly mitigate the adverse environmental impacts associated with conventional energy sources like coal and oil. This can enhance the environmental condition of Malaysia's tourist destinations, making them more appealing to visitors increasingly concerned with sustainability [134]. Additionally, clean energy sources are often found to be more efficient than conventional energy sources, thereby presenting a promising opportunity for tourism enterprises in Malaysia to effectively lower their energy expenses, thus enhancing the profitability and long-term sustainability of the tourism industry [135]. In addition to enhancing the tourism experience, using renewable energy sources has the potential to offer visitors a distinctive and environmentally conscious experience [136]. Ecotourism activities, such as hiking, camping, and wildlife observation, have the potential to be powered by renewable energy sources. This would enhance the authenticity of the visitor experience and contribute to the sustainability of such activities.

The study documented adverse linkage in the linear and nonlinear assessment regarding environmental sustainability impact on tourism development. Study findings advocate that reducing carbon emissions in the ecosystem enhances the environmental quality, intensifies tourism development, and contributes substantially to the economy. The negative linkage between carbon emission and tourism development is in line with the existing literature see, for instance Refs. [113,137,138]. The growth of tourism is highly reliant on natural, cultural, and social settings. Therefore, it is essential for tourism to either preserve or further improve the condition of the environment. Urban tourism, host-tourist interactions, and the production of tourist spaces for visiting urban areas with conflicting intentions, visiting attractions, and using facilities and services have various impacts on urban spaces and the economy. Urban tourism is the practice of traveling to and staying in urban areas to engage in activities typically associated with tourists. The study by Andriotis and Vaughan [139] discovered that seaside tourism has a modest influence on sustainable urban development, and this impact is sometimes regarded as an environmental concern. The study further argued that tourism development is not only an excessive and improper horizontal development of the city but also environmental pollution, most notably the pollution of water resources, which led to the city's excessive and improper horizontal development.

The symmetric and asymmetric assessment of FDI influence on tourism development revealed positive and statistically significant at a 1 % level, suggesting that the inflow of FDI prompts tourism activities in the economy. Our study findings are in line with existing literature [[140], [141], [142]]. Foreign direct investment (FDI) in the tourist industry is vital for its continued growth [143], particularly in nations still building their tourism industry. Foreign direct investment makes it possible for host nations to become connected to worldwide tourism networks, which will result in a rise in the Number of visitors visiting the country and an increase in the amount of money generated from tourism-related activities [144]. Foreign direct investment (FDI) may catalyze tourism, as pointed out by Amin, Al Kabir [145]. Investors must go to the host nation to gather knowledge and resources that are not accessible in the home country or via official papers to mitigate the risks associated with the FDI process and guarantee profitability. Increases in both FDI and tourism are sometimes cited as evidence of a direct relationship between the two industries (i.e., new tourist attractions and venues). Foreign direct investment (FDI) focusing on exports and growing commerce raises consumers' and businesses' knowledge of products and services that may pique their interest in business and leisure trips [146]. Another viewpoint is that international tourist spending is a key factor in attracting foreign direct investment to advanced economies. Using Tobit's analysis, Sanford and Dong (2000) discovered a positive and statistically significant correlation between tourism and FDI in the United States and a direct causal link between the two. According to Sanford and Dong (2000), international tourists might learn about the business climate and opportunities in a foreign place via personal experience. Other research has looked at how foreign direct investment (FDI) and tourism interact in developing countries. Tang et al. (2007) identified a one-way causal correlation between FDI and tourism growth in China. Still, Willem and Nair (2006) found no significant association in a panel dataset of Caribbean nations.

The study highlights a significant finding regarding the positive correlation between education (EDU) and tourism development (TD). This finding sheds light on the vital role that human capital plays in promoting the growth of the tourism sector. The importance of this relationship is emphasized by the statistically significant long-term (π = 0.0542, p < 0.01) and short-term (π = 0.0312, p < 0.01) coefficients. These coefficients indicate a consistent positive impact of education on tourism development in Malaysia. This discovery aligns with the current body of literature and provides valuable insights into the role of education in stimulating tourism expansion. One of the most crucial ways education facilitates tourism development is by enhancing the caliber of services and experiences provided to travelers. A highly educated local workforce is more adept at delivering outstanding customer service, offering valuable cultural perspectives, and creating personalized visitor experiences. By implementing these measures, there is a potential for a substantial improvement in the overall satisfaction of tourists, which can lead to increased repeat visits, positive word-of-mouth recommendations, and extended duration of stay. Education is crucial in equipping individuals with essential communication skills, adaptability, and a profound comprehension of tourist preferences. Consequently, this empowers them to contribute towards fostering a dynamic and captivating tourism ecosystem.

Furthermore, the study aptly highlights the crucial role that educational institutions play in fostering the development of individuals who actively contribute to the progress and enhancement of the tourism sector. Educational institutions play a crucial role in meeting the evolving demands of the tourism industry by providing a range of training programs, courses, and degrees specifically tailored to the field. These offerings help cultivate a talent pool well-equipped with the necessary skills and knowledge to excel in various tourism-related roles. Individuals who have obtained higher education in hospitality, tourism management, and other relevant disciplines are more likely to possess the necessary expertise to spearhead innovation, establish sustainable practices, and assume leadership roles within the industry, thereby propelling it toward future success. The congruence between the study's findings and the previous research conducted by Refs. [[118], [119], [120], [121], [122]] serves to strengthen the reliability of the observed correlation between education and tourism development. Ensuring consistency across multiple studies bolsters the credibility of the findings and solidifies education as a dependable predictor of tourism growth. Nevertheless, it is crucial to acknowledge that the association between education and tourism development, as emphasized in the study, may be subject to diverse contextual factors. For example, the caliber of education, the availability of educational opportunities, and the alignment of educational programs with the tourism industry can all influence the degree to which education contributes to tourism development. Policymakers and educational institutions ought to engage in collaborative efforts to ensure that education is customized to effectively address the specific requirements of the tourism sector. This collaboration aims to enhance not only the quantity but also the quality of education provided.

The study's findings emphasize the crucial role of Information and Communication Technology (ICT) in enhancing tourism development in Malaysia. These insights are valuable contributions to both the tourism industry and academic discussions. The presence of positive coefficients in both the long-run (π = 0.1199, p < 0.01) and short-run (π = 0.0618, p < 0.01) contexts confirms the favorable influence of technological advancements on the expansion of the tourism industry. This statement aligns with the current body of literature. It presents a persuasive case for the significant impact of information and communication technology (ICT) on developing tourism experiences and operational practices. The study appropriately emphasizes the various ways in which ICT innovations have a positive impact on the development of tourism [40,85]. Implementing enhanced online booking systems facilitates the reservation process, thereby enhancing travelers' convenience and optimizing service providers' efficiency. Creating mobile applications that are easy for users to navigate serves the purpose of assisting tourists in their exploration of various destinations. Additionally, these applications enhance tourists' interactions with local attractions, ultimately resulting in an overall increase in their satisfaction. Moreover, the provision of immersive virtual tourism experiences presents a groundbreaking opportunity for travelers to actively interact with destinations before their physical arrival, thereby enticing them to embark on a personal visit and engage in exploration [40,58,85,116,136,141].

The congruence between the study's findings and the existing literature, which includes the works of [61,85,125,127] highlights the strength and reliability of the observed correlation between ICT and tourism development. The consistency observed across various studies is a robust basis for asserting that technological advancements are crucial in shaping the tourism landscape. Nevertheless, it is imperative to consider potential challenges and limitations despite the study's emphasis on the favorable impact of information and communication technology (ICT) on tourism development. In certain contexts, not all regions or demographics may have equal access to advanced technology [61,108]. This disparity in access could result in digital divides, which may restrict information and communication technology (ICT) benefits. Furthermore, achieving a delicate equilibrium between embracing technological advancements and safeguarding the genuine essence and distinctiveness of various destinations is imperative. Excessive dependence on technology can result in a decline in the richness of cultural encounters and interpersonal connections that contribute to the lasting impact of tourism experiences [115]. Moreover, although technology enhances the tourism experience, it should not be considered a standalone solution. The successful incorporation of ICT necessitates the presence of suitable infrastructure, comprehensive training programs, and robust support systems. These elements are crucial to guarantee a smooth and gratifying experience for tourists and local stakeholders [120,136].

## 6 Conclusion and implications

The motivation of the study is to investigate the effects of clean energy, environmental sustainability and inflow of FDI on tourism development in the Malaysian economy from 1990 to 2019. For empirical nexus assessment and coefficients detection, the study has implemented several econometrical tools, including novel Bayer and Hanck [97] combined cointegration test, symmetric and asymmetric ARDL following Pesaran, Shin [62] and Shin, Yu [109], Toda and Yamamoto [128]- causality and Fourier TY causality. The key findings of the study are as follows.

The study underscores the significance of clean energy, foreign direct investment (FDI), education, and information and communication technology (ICT) in fostering tourism growth in Malaysia. The findings indicate that adopting renewable energy sources can improve tourist destinations' environmental state and reduce energy costs for tourism enterprises. Foreign Direct Investment (FDI) plays a crucial and influential role in attracting visitors and generating revenue from various tourism-related activities. Education plays a pivotal role in enhancing the quality of services and experiences offered to tourists, while ICT innovations further augment convenience, interaction, and overall satisfaction for travelers. Nevertheless, it is crucial to acknowledge contextual factors and potential obstacles, such as disparities in access and preserving the genuine nature of destinations. These factors collectively contribute to the sustainable and progressive development of the tourism industry in Malaysia.

On policy suggests, considering the empirical coefficients and directional association, the study offered the following policy development targeted tourism development in Malaysia. First, Clean energy integration acts as a catalyst in thriving the prospect of tourism development; thus, it is advocated for formulation and effective implementation of energy policy favoring clean energy inclusion in the economy instead of fossil fuel. Second, Controlled environmental protection by lowering carbon emissions by promoting energy and operation efficiency. A study suggested that the Malaysian economy has to open an avenue for environmental and technological innovation by offering a conducive ambiance and incentives for the industry. Third, Foreign participation in the form of FDI allows capital adequacy and industrial development with technological knowledge sharing. However, the impact of FDI on tourism development is obvious in the form of infrastructural development; thus, the study asked the Malaysian government to offer an amicable and conducive investment ambiance with financial efficiency and intermediation.

## Ethical approval

Not applicable.

## Consent to participate

Not applicable.

## Consent to publish

Not applicable.

## Funding

This paper was supported by the 10.13039/100020566Institute of Advanced Research (IAR), 10.13039/100019458United International University (UIU). Reference: IAR-2023-PUB-039.

## Availability of data and materials

All the data considered for the study are publicly available. Please see World Development Indicator (WDI), and TC data published by the World Bank, World Governance Index (WGI).

## CRediT authorship contribution statement

**Md Qamruzzaman:** Conceptualization, Data curation, Formal analysis, Funding acquisition, Writing – original draft, Writing – review & editing.

## Declaration of competing interest

The authors declare that they have no known competing financial interests or personal relationships that could have appeared to influence the work reported in this paper.
